# Adenoma of the nipple projecting out of the nipple: curative resection without excision of the nipple

**DOI:** 10.1186/1477-7819-12-91

**Published:** 2014-04-10

**Authors:** Takaaki Fujii, Reina Yajima, Hiroki Morita, Satoru Yamaguchi, Soichi Tsutsumi, Takayuki Asao, Hiroyuki Kuwano

**Affiliations:** 1 Department of General Surgical Science, Graduate School of Medicine, Gunma University, 3-39-22 Showa-machi, Maebashi, Gunma 371-8511, Japan; 2 First Department of Surgery, Graduate School of Medicine, Dokkyo University, Tochigi, Japan

**Keywords:** Adenoma of the nipple, Breast tumor, Nipple preservation

## Abstract

**Background:**

Adenoma of the nipple is a rare breast tumor that is often mistaken clinically for Paget’s disease and misinterpreted pathologically as invasive ductal carcinoma.

**Case report:**

We report herein a distinctive case of adenoma of the nipple projecting out of the nipple. In the current case, we were able to perform curative resection through a periareolar incision similar to a microdochectomy without excision of the nipple. The diagnosis of adenoma of the nipple was confirmed histopathologically.

**Conclusion:**

Although the tumor was found on top of the nipple, curative tumor resection without excision of the nipple was possible and the nipple was completely preserved. Adenoma of the nipple is a benign tumor, and thus the diagnosis of adenoma of the nipple must be confirmed so that unnecessary surgery can be avoided.

## Background

Adenoma of the nipple is a rare breast tumor that can present various histologic features [1-4]. The most common symptom is erosion of the nipple and nipple discharge, followed by induration or tumor formation at the nipple [5,6]. Adenoma of the nipple is an uncommon lesion that is often mistaken clinically for Paget’s disease and misinterpreted pathologically as ductal carcinoma [1-3,6-9]. However, adenoma of the nipple is a benign lesion that can be successfully treated by simple surgery. We report herein a distinctive case of adenoma of the nipple projecting out of the nipple. Although the tumor was found on top of the nipple, curative tumor resection without excision of the nipple was possible and the nipple was cosmetically preserved.

## Case presentation

**Figure 1:**
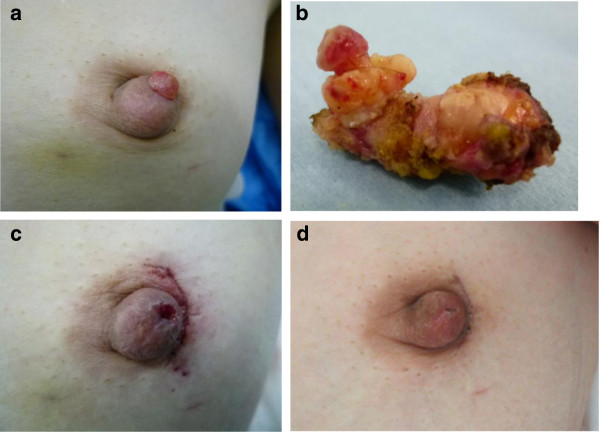
**Preoperative and postoperative images. (a)** Local finding of the left nipple: a red granulation-like tumor on top of the nipple. The mass was soft and fragile and bled easily. **(b)** The resected specimen contained a solid mass continuing with a projected mass. **(c)** At the end of the operation: curative excision of the lesion through a periareolar incision under local anesthesia. The mass was resected at a latex duct opening on top of the nipple, similar to a microdochectomy. **(d)** One month postoperatively.

**Figure 2:**
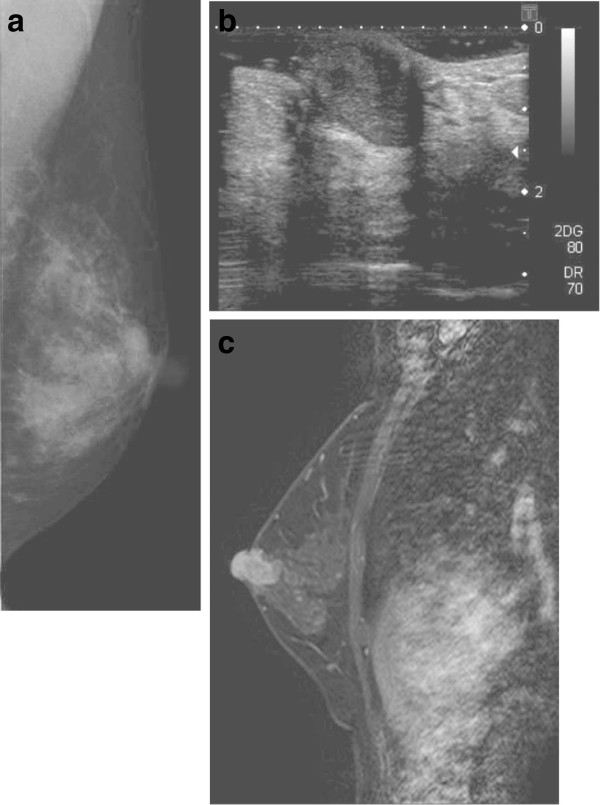
**Mammography, sonography and MRI images. (a)** Mammography revealed a well-defined, oval-shaped nodule with uniform density and no microcalcification. **(b)** Sonography and **(c)** MRI revealed that the mass under the nipple was continuous with the top of the nipple, showing adenoma of the nipple projecting out of the nipple. MRI, magnetic resonance imaging.

A 41-year-old Japanese woman presented with a red granulation-like tumor on top of the nipple of the left breast (Figure 1a). The mass was soft and fragile and bled easily. The patient had noticed the mass 3 months prior to her visit, and during that interval the mass had increased in size and the patient had observed a nipple discharge. Mammography revealed a well-defined oval-shaped nodule with uniform density and no microcalcification (Figure 2a). Sonography revealed an oval, well-demarcated hypoechoic solid mass under the nipple of the left breast (Figure 2b). Magnetic resonance imaging (MRI) demonstrated that the mass under the nipple was continuous with the top of the nipple, showing adenoma of the nipple projecting out of the nipple (Figure 2c). There was no evidence of axillary lymphadenopathy. Nipple discharge showed papillary clusters of epithelial cells. A core needle biopsy revealed a papillary proliferation of glandular structures suggestive of a benign adenomatous process. The patient received local curative excision of the lesion through a periareolar incision under local anesthesia. The mass was enucleated retrograde from the nipple and resected at a latex duct opening on top of the nipple, similar to a microdochectomy (Figure 1b). Curative resection without excision of the nipple was possible, and the nipple was cosmetically preserved (Figure 1c,d).

**Figure 3:**
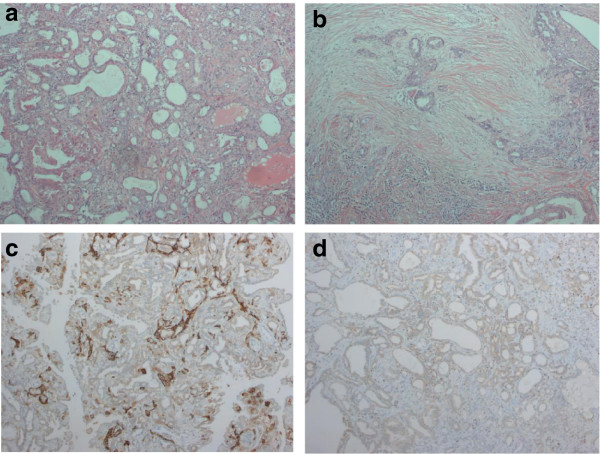
**H & E staining and immunohistochemical staining using CD10 and p63. (a)** H & E staining revealed papillary proliferation of epithelial cells. **(b)** Proliferation of glands showed a pseudoinvasive pattern. Immunohistochemical staining using **(c)** CD10 and **(d)** p63 was performed to confirm the presence of myoepithelial cells in neoplastic ducts. H & E, hematoxylin and eosin.

The histological evaluation revealed a papillary tumor composed of relatively large epithelial cells with clear nuclei and a nuclear fission image, but there was little increase in nuclear chromatin. The tumor cells were in two layers with some apocrine metaplasia (Figure 3). Myoepithelial cell staining also revealed myoepithelial cells arranged in an orderly manner. The papillary tumor was accompanied by moderate fibrosis, and a pseudoinfiltrative pattern was observed. Neither malignancy of the proliferative cells nor invasion was observed. These findings were compatible with adenoma of the nipple. Follow-up of the patient remained uneventful for 14 months.

## Discussion

Adenoma of the nipple is an uncommon benign tumor of the breast. It was first described by Jones [3] and occurs most often in 40- to 50-year-old patients [2,6]. The common clinical symptoms are nipple discharge and erosion or ulceration, in addition to a palpable tumor below the nipple [5,6]. Our case showed a unique palpable tumor under the nipple that was continued with a red granulation-like tumor on top of the nipple. In the World Health Organization (WHO) classification of breast tumors established in 2003 [10], adenoma of the nipple is defined as a compact proliferation of small tubules lined by epithelial and myoepithelial cells, with or without proliferation of the epithelial component, around the collecting ducts of the nipple [7]. The Japanese Breast Cancer Society defined adenoma of the nipple as a tumor developing papillary or solidly in the lactiferous duct of the nipple or just under the areola [6].

This is a rare breast tumor that can present various histologic features. The present case showed a combination of epithelial hyperplasia and sclerosing adenosis with pseudoinvasive features. Adenoma of the nipple is a completely benign tumor, but it is difficult to distinguish from ductal carcinoma in cases showing a marked invasion-like appearance or aggressive proliferative growth of intraductal tumor cells. Confirmation of the presence of two distinct layers of myoepithelial cells in neoplastic ducts is thought to be the most important finding for distinguishing adenoma from ductal carcinoma [1-3,7,11,12]. Immunohistochemical staining using CD10, p63 or α-smooth muscle actin can be useful for confirming the presence of myoepithelial cells in neoplastic ducts (Figure 3) [7,11,12]. The diagnosis of adenoma of the nipple must be confirmed so that unnecessary surgery can be avoided.

Since adenoma of the nipple is a completely benign tumor, we believe a limited surgical excision is the appropriate treatment. Although there are previous reports of total excision of the nipple and the areola with an underlying wedge of the breast and a complete resection of the nipple in patients with larger lesions, these procedures seem to be overly aggressive for benign disease [2,5,8]. In the present case, we were able to perform curative resection through a periareolar incision without excision of the nipple. The mass was resected by a method equivalent to microdochectomy. The nipple was completely preserved without the necessity for nipple reconstruction (Figure 1c,d).

## Conclusion

In conclusion, we have reported a rare case of nipple adenoma projecting out of the nipple. In our case, a limited local excision allowed us to preserve the nipple without remnant tumor. It is important for clinicians to be aware of the presence of this benign tumor so they can select appropriate treatment.

## Consent

Written informed consent was obtained from the patient for publication of this Case report and any accompanying images. A copy of the written consent is available for review by the Editor-in-Chief of this journal.

## Abbreviations

H & E: hematoxylin and eosin; MRI: magnetic resonance imaging; WHO: World Health Organization.

## Competing interests

The authors declare that they have no competing financial interests.

## Authors’ contributions

TF designed the study, provided the collection of the data of the patients and wrote the manuscript; RY, HM, SY, ST, TA provided the collection of all the human material; HK involved in editing the manuscript. This manuscript was read and approved the submission by all coauthors.
